# The effects of singing interventions on quality of life, mood and levels of agitation in community-dwelling people living with dementia: A quantitative systematic review

**DOI:** 10.1177/14713012241273837

**Published:** 2024-08-15

**Authors:** Megan Polden, Thomas Faulkner, Carol Holland, Kerry Hanna, Kym Ward, Faraz Ahmed, Heather Brown, Hazel Barrow, Jeanette Main, Stella Mann, Steve Pendrill, Clarissa Giebel

**Affiliations:** Department of Primary Care & Mental Health, 4591 University of Liverpool, UK; NIHR Applied Research Collaboration North West Coast, UK; Health Research, 4396 Lancaster University, UK; Department of Primary Care & Mental Health, 4591 University of Liverpool, UK; 4586 Mersey Care NHS Trust, UK; Health Research, 4396 Lancaster University, UK; Lyrics and Lunch Charity, UK; School of Health Sciences, 4591 University of Liverpool, UK; NIHR Applied Research Collaboration North West Coast, UK; 130122 The Brain Charity, UK; Health Research, 4396 Lancaster University, UK; Health Research, 4396 Lancaster University, UK; NIHR Applied Research Collaboration North West Coast, UK; NIHR Applied Research Collaboration North West Coast, UK; Lyrics and Lunch Charity, UK; NIHR Applied Research Collaboration North West Coast, UK; NIHR Applied Research Collaboration North West Coast, UK; Lyrics and Lunch Charity, UK; Department of Primary Care & Mental Health, 4591 University of Liverpool, UK; NIHR Applied Research Collaboration North West Coast, UK

**Keywords:** singing, dementia, music-based interventions, community-dwelling

## Abstract

**Background and Aims:** Music-based interventions have been found to benefit people living with dementia and have positive impacts on cognition and well-being. Most people with dementia live in the community and compared to people with dementia in residential care often have less access to music-based interventions. There are many forms of music interventions and singing has shown particular promise; in the realm of music interventions. It is important to determine what aspects of music interventions yield the most benefits for people with dementia. This review aimed to synthesise evidence on the impacts of singing interventions on quality of life, mood and neuropsychiatric symptoms for community-dwelling people with dementia. **Methods:** We systematically searched three electronic databases (PsycINFO, MEDLINE and Web of Science) for studies reporting on singing interventions with community-dwelling people with dementia. Studies were eligible for inclusion if they reported on a singing intervention with people living with dementia that included an outcome measure of quality of life, mood or agitation. Fourteen publications were identified and included in this review, with a total of *n* = 361 people with dementia. **Results:** Despite some inconsistencies across the literature, evidence suggests that singing interventions led to an improvement in mood and a reduction in agitation levels in people living with dementia. There was no strong evidence to suggest that singing interventions led to significant improvements in quality of life. **Conclusions:** This review highlights the potential of singing interventions as an effective psychosocial intervention for community-dwelling people with dementia. For key developments in this area, we urge that future studies include a control group where possible which will allow for more robust examinations of singing interventions and allow intervention effects to be distinguished from general deterioration in dementia symptoms over time.

## Introduction

It is estimated that over 55 million people are living with dementia worldwide (World Health Organisation, 2023). Dementia is not a part of normal ageing (Fiest et al., 2016) and is a group of major neurocognitive disorders for example Alzheimer’s disease, and vascular dementia. It is diagnosed when one or more cognitive domains are impaired such as executive functioning, inhibitory control, language, memory and learning according to the The Diagnostic and Statistical Manual of Mental Disorders, Fifth Edition (Guze, 1995), alongside disruptions to daily living. This is often assessed using tasks such as activities of daily living and instrumental activities of daily living (Pashmdarfard et al., 2020) that examine people’s ability to perform everyday tasks such as managing finances and medication, preparing a hot meal, and getting dressed. The cognitive deteriorations experienced in dementia have profound effects on people’s quality of life and ability to perform everyday tasks (Banerjee et al., 2006; Giebel et al., 2017). Changes in cognitive and emotional functioning within dementia often lead to behavioural and psychological changes, including, anxiety, depression, aggression, agitation, hallucinations and culturally inappropriate behaviours (Cagnin et al., 2012; Kwak et al., 2017). Depression is a common comorbidity of dementia with a reported prevalence of between 14-50% (Javadpour et al., 2017; Knapskog et al., 2014). Symptoms of depression in people with dementia include loss of appetite, irritability, loss of interest in activities, difficulty falling asleep, anxiety and suicide ideation (Alexopoulos et al., 1988). Impaired cognitive functioning is associated with a higher incidence of depressive symptoms, with depressive symptoms linked to poorer health outcomes in people with cognitive impairment (Chan et al., 2008). A systematic review revealed that pharmacological interventions were a common treatment for managing depressive symptoms and behaviours such as agitation in people with dementia (O’Donnell et al., 2022).

Pharmacological approaches to manage behavioural symptoms in people with dementia include antipsychotics, anxiolytics, hypnotics and antidepressant medications (Gustafsson et al., 2013). Although pharmacological interventions in some cases will improve behavioural symptoms, they are also often accompanied by negative side effects including increased occurrence of strokes (Maidment et al., 2018) and increased risk of mortality (Tampi et al., 2016). Antipsychotics may also worsen cognition and lead to a lower quality of life (Ballard & Corbett, 2010; Maidment et al., 2016). Due to the multiple negative side effects of pharmacological interventions, there is a strong need for effective non-pharmacological interventions to reduce and help manage behavioural symptoms of dementia (Moreno-Morales et al., 2020). The National Institute for Health and Care Excellence (NICE) guidelines recommend non-pharmacological interventions such as cognitive stimulation therapy, cognitive rehabilitation or occupational therapy to support functional ability and activities to promote well-being for people with dementia (National Institute for Health and Care Excellence, 2018).

Whilst not included in National Institute for Health and Care Excellence guidance yet, music therapy and music interventions have shown potential as an effective non-pharmacological intervention that can have a wide range of benefits for both people with dementia and their carers (Kelly et al., 2021; McDermott et al., 2013; Moreno-Morales et al., 2020; Raglio et al., 2012). Music therapy and interventions have been shown to improve mood, regulate emotion and reduce stress in people with dementia (Lee, Allison, O'Neill, Punch, Helitzer, Moss et al., 2022; Davidson & Almeida, 2014) and older adults (De Witte et al., 2022). It is theorised that music interventions lead to positive impacts via several factors including cognitive and neurological activation and stimulation (Sarkamo et al., 2008), memory activation (Cuddy et al., 2012), psychological and emotional impacts such as mood regulation and emotional expression (McCreedy et al., 2021), social interaction through verbal and non-verbal communication (Ridder et al., 2013), reduction in challenging behaviour and agitation and physiological effects through stress reduction and lower cortisol levels (Thaut et al., 2014). Theories suggest that these factors in combination lead to positive impacts and benefits for people living with dementia and their carers.

In recent years, research has focused on the specific aspects of music interventions that yield the best outcomes for people with dementia by comparing passive and active elements (Dowson & Schneider, 2021; Lam et al., 2020; Li et al., 2015). A systematic review of multiple variations of passive and active music interventions for people with dementia in residential care identified singing as a key element for change and substantial improvements in mood and reductions in behavioural symptoms (McDermott et al., 2015). Further evidence from a randomised control trial suggests that singing interventions that actively engage participants produce greater mood and quality of life improvements compared to more passive music listening interventions (Cho, 2018; Li et al., 2015). In addition, a study by Cheung et al. (2018) found greater improvements in memory and depressive symptoms in people with dementia following an active intervention including music with movement compared to a passive music intervention of music listening, highlighting the clear distinction in benefits following various types of music interventions. These studies indicate that singing and other forms of active engagement in music interventions may be key elements to have positive impacts on people with dementia. However, it should be noted that there is mixed evidence in the literature with a systematic review finding that music listening had a greater effect on people with dementia when compared with singing interventions (Moreno-Morales et al., 2020). This variation in findings may be a result of variations in dementia severity as some studies have suggested that active music interventions may be more beneficial in early to moderate stages of dementia and passive music interventions more beneficial in more advanced stages of dementia (Baker et al., 2022).

It is clear that there is a range of elements employed in music therapy and music interventions and singing is one particular element that evidence suggests may have positive impacts on people with dementia. Singing enhances neurological stimulation by combining music, language and instinctive human behaviour (Jeffries et al., 2003). Singing has been linked to increased cognitive functioning and improved memory performance as it engages and utilises brain pathways other than those active in plain speech (Carruth, 1997). This neurological stimulation can produce multiple benefits for people with dementia (Mansens et al., 2018; Satoh et al., 2015). Singing interventions, that are performed in a group format, have also been shown to improve social interactions between people with dementia and carers and reduce levels of agitation (Lin et al., 2011; Unadkat et al., 2017). Compared to other forms of active music interventions such as instrument playing, dancing or songwriting, singing offers a more accessible form of active music intervention for people living with dementia often including fewer barriers to engagement. In addition, singing interventions often require less equipment and set up compared to other forms of active music interventions aiding its practicality and ability to be widely delivered and cost-effective.

Multiple literature reviews have demonstrated the effects of music interventions on dementia symptomology (Ueda et al., 2013; Moreno-Morales et al., 2020; Lam et al., 2020; Soufineyestani et al., 2021); however, in general, the majority of previous reviews have mainly focused on studies in residential care settings as opposed to community-based settings. For example, Soufineyestani et al. (2021) conducted a review using a meta-narrative method and found that in most cases, music could be used as a safe and cost-effective non-pharmacological approach for dementia treatment. However, this review included mainly studies conducted in residential care and due to this, the impacts of music interventions specifically on community-based people living with dementia is unclear. The majority of these reviews do not distinguish the context in which the music interventions are delivered (care homes or community settings) and also the differing populations due to these differing contexts in terms of dementia severity, which is likely to affect the outcomes and impact of the intervention.

A recent review conducted by Hofbauer et al. (2022) focuses specifically on music interventions for community-dwelling people with dementia and found immediate impacts on cognition, short-term impacts (lasting 1–4 months) on cognition, anxiety and pain, but longer-term impacts (lasting 6 months plus) were less apparent for outcome measures. This review included music therapies and interventions that employ different methods and both active and passive formats ranging from individual activities or group activities incorporating music listening, playing instruments and/or singing. These activities and formats can vary substantially and may have differing effects and levels of influence on dementia symptoms. In order to determine the aspects of music interventions that yield optimal benefits for community-dwelling people with dementia it is important to examine specific forms of music interventions such as singing interventions.

To our knowledge, only one recent scoping review has specifically focused on the impact of singing interventions on people with dementia (Dowson & Schneider, 2021), examining online singing interventions. To date, no review has been conducted exploring in-person community-based singing interventions and their impact on people with dementia. Research suggests differing effects and outcomes based on the type of music intervention (active or passive), different contexts (community or care home setting) and severity of dementia (early or late stages). Community-based people with dementia will likely experience differing benefits of singing interventions than those living in residential care home settings such as an increase in social networks, community support and improvements in relationships with unpaid carers. Considering that the vast majority of people with dementia live at home (Dickinson et al., 2017), it is important to examine the impacts of singing interventions specifically in community settings for people with dementia.

Differing impacts of singing interventions in community-based people with dementia compared to people in care homes may be driven by improvements in factors surrounding well-being such as mood, agitation and quality of life rather than cognitive impacts which may have more similar impacts in differing settings. The current review focuses on quality of life, mood and agitation as previous reviews have often focused on the cognitive impacts of singing interventions rather than specifically focusing on factors surrounding well-being. There is a gap in the existing literature and a need for a quantitative systematic review to examine the impact of community-based singing interventions on quality of life, mood and agitation in people with dementia and the current systematic review seeks to address this gap. This review will examine both short-term and longer-term reported impacts of singing interventions.

## Methods

For this review, a narrative synthesis approach (Popay et al., 2006) was utilised, and the PRISMA guidelines were followed (Moher et al., 2009). The review was registered on PROSPERO (CRD42023395907) before the review commenced.

### Eligibility

#### Participants

Studies were eligible for inclusion if participants with dementia had received a formal diagnosis and were living within the community and not in full-time residential care. Studies that included another clinical group (i.e. Parkinson’s disease) were still eligible for inclusion but only the data from the dementia group was used in this review.

#### Intervention condition

Only quantitative studies were included. Studies were required to have an ‘experimental’ condition (including ‘natural’ and quasi-experiments) or trial arm in which people with dementia, who lived in the community, took part in a singing intervention, or employed a pre-post-test design. In cases where outcome data were available at multiple post-intervention time points, data were extracted from the longest pre and post-periods in which the singing intervention was being attended versus not. Only active singing interventions were eligible for inclusion with interventions examining passive music interventions such as music listening excluded. As we aimed to examine in-person community-based singing interventions (e.g. day centres, community centres), studies examining online singing interventions or interventions based in residential care homes were not included. Studies that included another intervention component alongside the singing intervention such as dancing or movement therapy were not included unless the study included a singing-only condition and in this case, the singing-only trial arm was included.

#### Comparator condition

Experimental studies were required to have a control condition or trial arm in which participants did not attend a singing intervention or another type of intervention. Pre-post-test design studies did not require a control group.

#### Outcome

As we aimed to examine the effect of singing interventions on quality of life, mood and levels of agitation, at least one measure of quality of life, mood or agitation was required to have been measured in the study, including the Dementia Quality of Life Scale (DEMQOL and DEMQOL-Proxy) (Smith et al., 2005), Quality of Life Scale (Flanagan, 1978), Hospital Anxiety and Depression Scale (Zigmond & Snaith, 1983), Quick Inventory of Depressive Symptomatology (Rush et al., 2003), and Neuropsychiatric Inventory Questionnaire (Kaufer et al., 2000). Eligible outcome measures were restricted to quantitative standardised assessment tools with self-assessment tools or assessments completed by proxy eligible for inclusion.

### Search strategy

We searched PsycINFO, MEDLINE and Web of Science from 01/03/2023 to 09/03/2023. The search string was developed for our population of interest (people living with dementia) and our intervention (singing interventions) including any relevant dictionary terms across our included databases, as well as free-text search terms identified from relevant articles (see appendix 1 for full search teams) Searches were restricted to articles published in English or German and there were no restrictions on publication dates. Thesis and conference abstracts were excluded. OSF (including PsychArxiv) *Medrxiv and SSRN* pre-print archives were also searched for unpublished studies. The online programme CADIMA was used to manage search outputs and screen articles (Kohl et al., 2018). For the electronic database searches, after the removal of duplicates, two researchers (MP and TF) independently completed title and abstract screening and full-text screening. One researcher (MP) searched the Open Science Framework pre-print archive and Social Science Research Network pre-print servers and a second researcher (TF) independently checked for eligibility for inclusion. Forwards and backward citation screening were conducted for all eligible articles (MP) and any identified articles confirmed for eligibility by a second researcher (TF). Any instances of disagreement between the two researchers (MP and TF) were resolved through discussion. Updated searches were conducted on 26/02/2024 using the original search strategy but limited to articles published in 2023 to 2024 to ensure eligible articles published after the initial searches were included in this review.

### Data selection and extraction

Two researchers (MP and TF) independently extracted information from all articles deemed to meet the inclusion criteria. In addition to bibliographic information, the following information was extracted: Country the study was conducted in, setting (i.e. community hall, daycare etc), participant sample and characteristics (e.g. gender, age, dementia severity), the primary quality of life, mood or agitation assessment, number of participants, intervention delivery (e.g. number of sessions, length of sessions, the period intervention was delivered), control group, study design (experimental or pre-post), outcome measure type (mood, quality of life or agitation), outcome measure time-period (e.g. for pre-post designs when the outcome was measured at baseline and then at follow-up), outcome measure data (e.g. Mean and standard deviation or standard error, p values and effect sizes).

### Risk of bias

We assessed the risk of bias using the Mixed Methods Appraisal Tool (MMAT) which has criteria for 5 study designs: qualitative, quantitative randomized controlled, quantitative nonrandomized, quantitative descriptive, and mixed methods (Hong et al., 2018). Each study category contained 5 criteria with a point given for each criterion met, resulting in a score ranging from 0-5 with 5 indicating a lower risk of bias. Scores of 0-2 were considered as high, scores of 3-4 were considered as moderate and a score of 5 was considered as low risk of bias. The risk of bias assessments were completed independently by two researchers (MP and TF) and differences were resolved through discussion.

## Results

### Study selection

**Figure 1.**
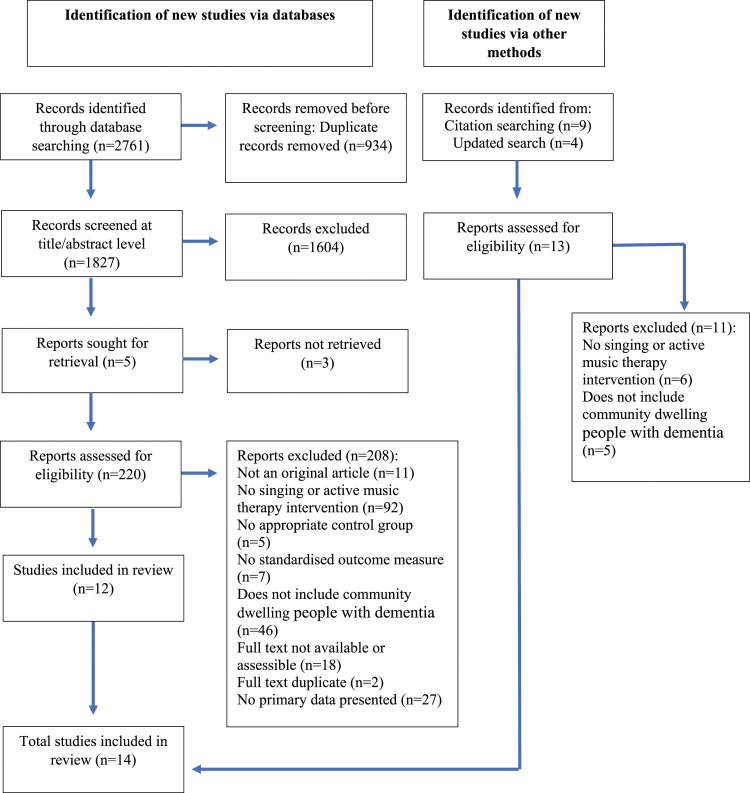
PRISMA Chart (Page et al., 2021).

Our search strategy identified 2,761 records and after the removal of duplicates, 1,827 were screened during the title and abstract screening process (Figure 1). Following this stage, 220 records were included in the full-text screening. After applying the inclusion and exclusion criteria in the full-text screening, we identified 12 publications eligible for inclusion in this review. A further article was identified during forward and backwards citation screening. Another article was identified during an updated search (screening articles published between 2023 to 2024) giving a total of 14 articles included in this review.

In this review we opted for a narrative synthesis (Popay et al., 2006) due to the heterogeneity in the intervention’s delivery and in some cases, the data required for a meta-analysis not being available/reported. In addition, due to the direction of effects varying for some outcome measures, this may have led to misleading results from a meta-analysis approach (Deeks et al., 2019). Results are synthesised below based on outcome measures (quality of life, mood, agitation).

### Characteristics of included studies

Studies included in this review originated from Australia, Brazil, Canada, Finland, France, Germany, Italy, Japan, Norway, Spain, Taiwan, the UK and the US (see Table 1). Of the 14 included studies, 10 were quasi-experimental (pre vs post design with no control group) (72%), one was experimental (7%), and three were randomised control trials (RCTs) (21%). Eight of the music interventions were delivered by music therapists, two by researchers, and four by professional musicians. The majority of interventions were group-based (*n* = 10), with a minority delivered in a one-to-one setting (*n* = 4). The majority of studies (*n* = 12) included people with various dementia subtypes such as Alzheimer’s Disease, vascular dementia, mixed dementia and other dementia types, although two studies only included people with Alzheimer’s Disease. Across all studies, 361 people with dementia were included. None of the included studies reported the ethnic background of participants. Of the 14 studies, eight assessed quality of life as an outcome measure (Table 2), seven assessed mood (Table 3), and ten assessed agitation (Table 4).

**Table 1.** Summary of included studies.

Authors (years)	Country study conducted	Relevant sub-sample	Setting of intervention	Description of intervention	Design	Outcome Measure
Aleixo et al, 2022	Brazil	*N* = 13 Dementia Type: AD (10), VaD ( 2) and MD (1) Severity: CDR mild (11), moderate (2), dementia stage Age 84±3.16, 100% female	Community based active music therapy group	Once weekly over a 12-week period in 60-minute sessions	Quasi-experimental, within-participants	Agitation and quality of life
Brotons and Marti, 2003	Spain	*N* = 14 Dementia Type: All AD Severity: Stages 4 or 5 of global deterioration scale Age 75.67±3.65, 36% female	Rural house where participants stayed for 12 days	10 group sessions with carer givers	Quasi-experimental, within-participants	Agitation
Camic et al, 2013	UK	*N* = 10 Dementia Type: AD (6) VaD (2), Mixed Dementia (1), Mild Cognitive Impairment (1) Severity: Mini Mental State Exam score 19±7.9 Age 75±6.70, 50% female	Community based group	group music-making and singing (professional choir leader) and met weekly over a period of 10 weeks for 90 minutes, including a break for refreshments	Quasi-experimental, within-participants	Agitation, quality of life and mood
Chen and Pei, 2018	Taiwan	*N* = 28 (EX *n* = 15, CT *n* = 13) Dementia Type: AD, VaD and other types Severity: CDR rating 0.5-2 EX: age 77.3±9.4, 60% female CT: age 77.3±10, 62% female	One to one sessions took place either at an activity room within the dementia specialty outpatient unit or at an activity room within the city district health centre	Participants attended training in eight weekly, individual, 60-min sessions. Each participant received the intervention across a period of 2 months	Randomised-control trial	Agitation
Dawudi et al, 2023	Germany	*N* = 19 Dementia Type: not specified Severity: mild-to-moderate dementia, mean Mini Mental State Exam score 16.42 (SD = 5.79) Age 76.95±8.18 52.6% female	Community based group	Seven mid-day weekly choir sessions lasting 1.5 hours each. Provided by a professional choir leader, accompanied by a piano player. Sessions were supported by a music therapist.	Quasi-experimental, within-participants	Agitation, quality of life and mood
Holden et al, 2019	USA	*N* = 18 Dementia Type: All dementia types Severity: mild-to-moderate (9), moderate-to-severe (9) Age 76.9±10, 39% female	Individual music therapy in person with dementia’s home	Active music therapy for 60 minutes, 6-week period, 1 session per week	Quasi-experimental, within-participants	Agitation and quality of life
Jennings and Vance, 2002	USA	*N* = 16 Dementia Type: AD Severity: not reported Age 78.80±3.91, 87.5% female	Adult day-care centre	30-minute active music class once per week for 4 weeks	Quasi-experimental, within-participants	Agitation
Madsø et al, 2022	Norway	*N* = 13 Dementia Type: AD (11), vascular dementia (2) Severity: CDR very mild (1), mild (5), moderately severe (5) Age 79.82±5.27, 69% female	Home based one to one sessions	30 minutes music sessions weekly for 10 weeks	Quasi-experimental, within-participants	Agitation, quality of life and mood
McDowell et al, 2023	Canada	*N* = 33 Dementia Type: AD (29), other dementia (4) Severity: Mini Mental State Exam score at intake was 20.6 (SD = 5.7, range = 10–29) Age 79.1±8, 55 % female	Community based choir group held at two local churches	Three choir seasons (each spanning approximately 3.5-months) across a 1.5-year period. During the choir seasons, participants met weekly for a two-hour choir session that consisted of a warm-up, song rehearsal, and concluded with a 30-minute period of socialization	Quasi-experimental, within-participants	Mood
Pongan et al, 2017	France	*N* = 31 Dementia Type: AD Severity: mild AD Age 78.8±7.43, 74% female	Community based group singing	Choir singing and song learning, 2 hour singing group once per week for 12 weeks	Experimental, within-participants	Quality of life, mood
Raglio et al, 2016	Italy	*N* = 4 Dementia Type: VaD (2), frontotemporal dementia (1), AD (1) Duration of disease mean: 6.75 years Age 78.25, 25% female	Group community-based active music therapy	12 active music therapy 40-minute sessions twice per week for 6 weeks conducted by a trained music therapist	Quasi-experimental, within-participants	Mood, agitation
Sarkamo et al, 2013	Finland	*N* = 89 (EX = 30, CT = 29) Dementia Type: AD (26), VaS (12), MD (7), Other (10) Severity: CDR EX = 1.0, CT = 1.1 EX: Age 78.5±10.4, 53% female CT: 78.4±11.6, 60% female	Group community singing group	10-week group-based music coaching program, which included either singing sessions (SG) or music listening sessions. The singing and music listening sessions were held weekly (1.5 hr/session) at each centre for a group of 10 participants (5 people with dementia, 5 caregivers), and they were led by a trained music teacher or music therapist	Randomised-control trial	Mood, quality of life
Tamplin et al, 2018	Australia	*N* = 12 Dementia Type: All type dementia Severity: Mini Mental State Exam score between 10 and 26 Age 77.9±9.9, 58% female	Community singing group	20-week therapeutic group singing intervention, once weekly for 2-hours. Sessions were co-facilitated by two trained music therapists. The intervention incorporated vocal warm ups, singing familiar songs, learning new songs, and opportunities for social interaction	Quasi-experimental, within-participants	Quality of life
Satoh et al. (2015)	Japan	*N* = 20 (EX = 10, CT = 10) Dementia Type: AD Severity: MMSE score EX = 19.1, CT = 20.9 EX age 78.1±7.0, 60% female CT age 77±6.1, 80% female	Group held in hospital once per week	Singing training at a hospital once a week for 6 months (24 sessions). Each session was 1 hour. The sessions consisted of the following sections: voice training performed for 15 minutes, a 15-minute review of the former session, for 20 minutes participants practiced familiar songs, last 10-minutes was so-called recreation time.	Experimental, within-participants	Agitation

*Note.* AD = Alzheimer’s Disease, MMSE = Mini mental state examination, EX = Experimental condition, CT = Control condition, VaD = Vascular dementia, CDR = Clinical dementia rating, MD = mixed dementia.

**Table 2.** Results of outcome measure: Quality of life.

Authors (years)	Measures	Measurement time points	Main findings
Aleixo et al., 2022	QOL-AD, QOL informant	T0 versus T1 (12 weeks)	T0 versus T1 • For the QOL-AD, there were no significant differences between T0 (34.84 ± 6.26) versus T1 (35.20 ± 4.51) for QOL score (*p* = 0.69) • For the QOL informant there were no significant difference between T0 (30.23 ± 7.13) versus T1 (29.69 ± 6.71) (*p* = 0.54)
Camic et al., 2013	DEMQOL	T0, T1 (10 weeks), T2 (T1 + 10 weeks)	T0 versus T1 versus T2 • No significant change in DEMQOL score between T0 (90.67 ± 13.28), T1 (80.27 ± 8.17) or T2 (81.95 ± 16.70)
Dawudi et al., 2023	QOL-AD	T0 versus T1 (2 weeks after final session)	T0 versus T1 • No significant change in QOL-AD score between T0 (37.18 ± 3.86) to T1 (38.12 ± 5.16) *p* = .234
Holden et al., 2019	QOL-AD, QOL- informant	T0, T1 (6 weeks), T2 (T1 + 6 weeks)	T0 versus T1 versus T2 • For the QOL-AD patient no significant changes after T1 (*p* = 0.43) or T2 (*p* = 0.16) • For the QOL-AD informant no significant changes in score between T0 versus T1 (*p* = .84) or T0 versus T2 (*p* = .41)
Madsø et al., 2022	QOL-AD	T0 versus T1 (10 weeks)	T0 versus T1 • No significant change in quality of life scores from T0 (medium = 22) to T1 (medium = 26) (*p* = .72)
Pongan et al., 2017	EuroQol-5 dimensions	T0 versus T1 (12 weeks), T2 (12 weeks + 4 weeks)	T0 versus T1 versus T2 • Quality of life scores significantly improved post intervention (T0 = 9.65 ± 3.41, T1 = 9.86 ± 2.69, T2 = 8.75 ± 3.60), *p* = 0.002
Sarkamo et al., 2013	QOL-AD, QOL informant	T0, T1 (10 weeks), T2 (10 weeks + 24 weeks)	T0 versus T1 versus T2 • QOL-AD: only music listening had an effect on quality of life (*p* = 0.033) and the singing group did not show significant differences in QOL-AD scores at either time points • QOL informant showed similar pattern of results with no significant changes in QOL scores between time-points
Tamplin et al., 2018	QOL-AD, QOL informant	T0, T1 (midway through intervention, 10 weeks), T2 (20 weeks)	T0 versus T1 versus T2 • no statistically significant changes from T0, T1 and T2 on quality of life assessments however there were small effect sizes suggesting decreased quality of life from T0 versus T2 intervention (d = −0.20)

*Note.* T0 = baseline, T1 = timepoint 1, T2 = timepoint 2, DEMQOL = Dementia Quality of Life measure, QOL-AD = Quality of life- Alzheimer’s Disease.

**Table 3.** Results of outcome measure: Mood.

Authors (years)	Measures	Measurement time points	Main findings
Camic et al, 2013	Geriatric Depression Scale	T0, T1 (10 weeks), T2 (T1 + 10 weeks)	T0 versus T1 versus T2 • Significant increase in geriatric depression scale scores from T0 to T1 (T0 = 2.71 ± 2.30, T1 = 4.17 ± 3.31, T2 = 3.34 ± 3.50)
Dawudi et al, 2023	Geriatric Depression Scale	T0 versus T1 (2 weeks after final session)	T0 versus T1 • No significant change in geriatric depression scale score between T0 (2.50 ± 2.23) to T1 (2.28 ± 1.97) (*p* = .323)
Madsø et al, 2022	Visual Analog Mood Scale	T0 versus T1 (10 weeks)	T0 versus T1 • Visual analog mood scale scores found a reduction in negative emotions (*p* < .001) and an increase in positive emotions (*p* < .001) when comparing T0 and T1 assessments
McDowell et al, 2023	Positive and Negative Affect Schedule	Baseline (start of choral season) and then every 3–4 weeks during choral season (up to nine assessments per participant)	Significant decreases in negative effect were linked to improvements in cognitive functioning (*p* = 0.02)
Pongan et al, 2017	Geriatric Depression Scale	T0 versus T1 (12 weeks), T2 (12 weeks + 4 weeks)	T0 versus T1 versus T2 • The singing intervention group did not show significant changes between T0 (8.81 ± 35.98), T1(9.48 ± 6.39) and T2 (8.96 ± 6.40) for depressive symptoms (*p* = 0.10)
Raglio et al, 2016	Geriatric Depression Scale	T0, T1 (6 weeks), T2 (6 weeks + 4 weeks)	T0 versus T1 versus T2 • Due to small sample sizes (4 people with dementia) the study only reported mean values and did not conduct statistical testing. Mean values showed a reduction in depression scores between T0, T1 and at T2(follow up) (T0 = 14.5; T1 = 11; T2 = 11.75)
Sarkamo et al, 2013	Cornell-Brown Scale for Quality of Life in Dementia	T0, T1 (10 weeks), T2 (10 weeks + 24 weeks)	T0 versus T1 versus T2 • Compared to the control group (usual care) both the singing and music listening groups showed improved mood post-intervention (*p* = .001) • Effects on mood were no longer statistically significant at follow-up (*p* = .116)

*Note.* T0 = baseline, T1 = timepoint 1, T2 = timepoint 2.

**Table 4.** Results of outcome measure: Agitation.

Authors (years)	Measures	Measurement time points	Main findings
Aleixo et al, 2022	Neuropsychiatric inventory	T0 versus T1 (12 weeks)	T0 versus T1 • Slight decrease in neuropsychiatric inventory scores post intervention (T0 M = 8, T1 M = 3), although not statistically significant, *p* = 0.27
Brotons and Marti, 2003	Neuropsychiatric inventory, Cohen-mansfield agitation questionnaire	T0, T1 (10 days), T2 (12 days + 8 weeks)	T0 versus T1 versus T2 • Neuropsychiatric inventory scores were significantly lower at T1 (M = 5.42) and T2 (M = 17.18) compared to T0 (M = 23.58), *p* = 0.001 • Cohen-mansfield agitation questionnaire scores were significantly lower post intervention at timepoints T1 (M = 2.33) and T2 (M = 4.26) compared to T0 (M = 6.17), *p* = 0.003
Camic et al, 2013	Neuropsychiatric inventory	T0, T1 (10 weeks), T2 (T1 + 10 weeks)	T0 versus T1 versus T2 • Neuropsychiatric inventory scores were higher at T1 (16±10.9) and T2 compared to T0 (20.1±13.7)
Chen et al., 2018	Chinese community-version Cohen-Mansfield agitation inventory scale	T0 versus T1 (8 weeks)	T0 versus T1 • A significant effect favoured the intervention compared to the control condition for agitation scores (*p* < 0.01)
Dawudi et al, 2023	Neuropsychiatric inventory	T0 versus T1 (2 weeks after final session)	T0 versus T1 • No significant change in NPI score between T0 (15.68 ± 18.36) to T1 (12.72 ± 10.55) (*p* = .299)
Holden et al, 2019	Neuropsychiatric inventory	T0, T1 (6 weeks), T2 (T1 + 6 weeks)	T0 versus T1 versus T2 • Neuropsychiatric inventory scores were significantly lower post intervention at timepoints T1 (19.1 ± 11.7, *p* = 0.02) and T2 (18.6 ± 14.0, *p* = 0.04) compared to T0 (28.4 ± 16.2)
Jennings et al., 2002	Modified Cohen-Mansfield agitation inventory (assessed by each participant’s primary day care staff person)	T0, T1 (within 45 minutes to one hour after each music class)	T0 versus T1 • CMAI scores were significantly lower after music classes compared to T0 (*p* < .001) indicating a reduction in agitation and neuropsychiatric symptoms following the intervention
Madsø et al, 2022	Neuropsychiatric inventory	T0 versus T1 (10 weeks)	T0 versus T1 • Significant improvement in neuropsychiatric inventory scores from T0 to T1, *p* = .014
Raglio et al, 2016	Neuropsychiatric inventory	T0, T1 (6 weeks), T2 (6 weeks + 4 weeks)	T0 versus T1 versus T2 • An improvement in means of neuropsychiatric inventory scores from T0 to post intervention (T1), with these improvements sustained at a 1-month follow-up (T2) (T0 = 30.75; T1 = 28.50; T2 = 27.25) • Did no conduct statistical testing due to small sample size
Satoh et al. 2015	Neuropsychiatric inventory	T0, T1 (24 weeks)	T0 versus T1 • Significant decrease of neuropsychiatric inventory score post intervention (*p* = 0.042) indicating a reduction in agitation

*Note.* T0 = baseline, T1 = timepoint 1, T2 = timepoint 2.

### Outcome measure: Quality of life

Eight studies included quality of life as an outcome measure (Aleixo et al., 2022; Camic et al., 2013; Dawudi et al., 2023; Holden et al., 2019; Madsø et al., 2022; Pongan et al., 2017; Sarkamo et al., 2014; Tamplin et al., 2018). Of the eight studies only one found that attending a singing intervention increased quality of life (Pongan et al., 2017) which employed a RCT design while the other studies employing pre/post and RCT designs found no significant effect (Aleixo et al., 2022; Camic et al., 2013; Dawudi et al., 2023; Holden et al., 2019; Madsø et al., 2022; Sarkamo et al., 2014; Tamplin et al., 2018). When examining the mean values for studies without significant findings two studies showed a decrease in quality of life scores (Camic et al., 2013; Tamplin et al., 2018) and the others showed a minimal increase (Aleixo et al., 2022; Dawudi et al., 2023; Holden et al., 2019; Madsø et al., 2022; Sarkamo et al., 2014). Pongan et al. (2017), included people with dementia who were also experiencing chronic pain and this may have led to the variation in results seen here. The improvement in quality of life correlated with a reduction in chronic pain ratings and this may have indirectly impacted quality of life scores rather than the intervention itself.

Except for Sarkamo et al. (2014), studies not demonstrating an effect of the intervention on quality of life included small sample sizes (*n*<20). Pongan et al. (2017) had a larger sample size (*n* = 31 in the singing condition) which may have provided greater power to detect changes on quality of life measures. However, it may be the case that the quality of life measures may be too general to detect subtle changes in quality of life as a result of singing interventions and that subsection analysis on quality of life measures may be required to determine if certain aspects are improved over others (for example DEMQOL includes three subsections mood, memory and everyday life). Overall results indicate that singing interventions do not appear to have a significant impact on quality of life for people with dementia and further research is required with larger sample sizes for the impacts to be determined.

### Outcome measure: Mood

Seven of the studies examined mood or depressive symptoms as an outcome variable (Sarkamo et al., 2013; McDowell et al., 2023; Camic et al., 2013; Pongan et al., 2017; Madsø et al., 2022; Raglio et al., 2016, Dawudi et al., 2023). Of these seven studies, four found that the intervention significantly improved mood, three using pre/post designs and one using a RCT design (Sarkamo et al., 2013; McDowell et al., 2023; Madsø et al., 2022; Raglio et al., 2016). Sarkamo et al. (2013) found the effects were no longer significant at follow-up 6 months after the interventions end. Conflicting with this finding, Camic et al. (2013) using a pre/post design found higher levels of depressive symptoms post-intervention but this aligned with general deterioration within the participant sample. Similarly, Pongan et al. (2017) conducted a RDT and found no significant changes in mood and Dawudi et al. (2023) using a pre/post design also found no significant changes.

A distinguishing factor between these studies is that all but one implemented an intervention lasting 12 weeks or less, whereas McDowell et al. (2023) evaluated a longer-term choral intervention delivered in three 3.5-month blocks over the course of a year and a half. Additionally, McDowell et al. (2023) included 30 minutes of socialisation at the end of the choral sessions and this increased social aspect alongside the singing may be important for improvements in mood in people with dementia. Two studies that examined one-to-one singing interventions (Madsø et al., 2022; Raglio et al., 2016) found improvements in mood despite no group socialisation element and therefore it is unclear whether the inclusion of a socialisation period was the differing factor for improvements in mood in the McDowell et al. (2023) study. Across these six studies, the majority found that singing interventions improved mood for people with dementia despite a range of intervention delivery methods. This indicates that improvements in mood following singing interventions are fairly robust and not easily diminished due to external intervention delivery factors.

### Outcome measure: Agitation and neuropsychiatric Symptoms

Ten studies included neuropsychiatric symptoms as an outcome measure (Aleixo et al., 2022; Jennings & Vance, 2002; Bronton, 2003; Camic et al., 2013; Chen & Pei, 2018; Dawudi et al., 2023; Holden et al., 2019; Madsø et al., 2022; Raglio et al., 2016; Satoh et al., 2015). Studies overall showed fairly consistent results with seven studies showing significant reductions in neuropsychiatric symptoms post-intervention with the majority employing pre/post designs (Chen & Pei, 2018; Holden et al., 2019; Madsø et al., 2022; Raglio et al., 2016; Satoh et al., 2015; Jennings & Vance, 2002; Bronton, 2003). However, two pre/post studies showed no significant improvements in overall neuropsychiatric symptoms post-intervention but an improvement in anxiety (Aleixo et al., 2022; Dawudi et al., 2023) and one study showed an increase in neuropsychiatric symptoms (Camic et al., 2013).

Similar to McDowell et al. (2023), Satoh et al. (2015) examined a longer 6-month intervention and this longer period of intervention delivery time may be a key factor in the reduction of neuropsychiatric symptoms in people with dementia. However, Jennings & Vance (2002) and Bronton and Marti (2003) provided shorter interventions (4 weeks and 12 days) and found a reduction in neuropsychiatric symptoms and agitation. Unlike the other studies, Jennings & Vance (2002) compared levels of agitation within 45 minutes before and then shortly after each weekly session rather than comparing baseline assessments to post-intervention assessments after several weeks. This method may be more suitable to determine short-term effects on neuropsychiatric symptoms in people with dementia. Similar to the findings in Camic et al. (2013), deterioration of symptoms is likely to occur in people with dementia over a period of time and this may mask intervention effects and supports the need for the inclusion of control groups in study designs. In people with dementia, stability and a lack of deterioration of symptoms over time can also be meaningful and indicate a positive impact of the intervention.

Unlike other studies, Bronton and Marti (2003) delivered a more intensive music intervention with 10 sessions over 12 days. This delivery may be a contributing factor in the resulting short-term and longer-term (2-month follow-up) reductions in neuropsychiatric symptoms seen in this study. However, Satoh et al. (2015) conducted a longer period intervention over 6-months and found improvements and it is unclear from these studies what is the optimal way to deliver singing interventions to optimise a reduction in neuropsychiatric symptoms – that is, short intensive designs or longer durations.

Dawudi et al. (2023) found a non-significant reduction in neuropsychiatric symptoms however, this study also examined saliva cortisol levels as an indicator of stress before and after each choral session and found a significant reduction in cortisol after the session. Physiological measures may be less affected and sensitive to changes in dementia severity and a general deterioration of symptoms than standardised questionnaires. The findings from these studies indicate that singing interventions appear to have a positive impact on agitation and neuropsychiatric symptoms in people with dementia across both a range of intervention delivery methods and following shorter and longer intervention designs.

### Longer-term effects of singing interventions

It is important to establish whether any improvements in outcome measures are sustained beyond the end of the intervention and for how long. Of the 14 studies, six conducted follow-up assessments after the intervention’s end (Brotons & Marti, 2003; Camic et al., 2013; Sarkamo et al., 2013; Pongan et al., 2017; Raglio et al., 2016; Tamplin et al., 2018). Of these studies, three found that effects were sustained after the intervention with Bronton (2003) finding that levels of agitation were reduced two months post-intervention (relative to baseline), Pongan et al. (2017) found that improvements in quality of life were still present four weeks post-intervention and Raglio et al. (2016) found that improvements in neuropsychiatric inventory questionnaire scores and depression scores were sustained at a 1-month follow-up. It should be noted that Raglio et al. (2016) only reported mean values due to small sample sizes (4 people with dementia) and did not conduct statistical testing. As a result, for this study, it cannot be determined whether these results were statistically significant and should be interpreted with caution. In contrast, Sarkamo et al. (2013) found that the effects on mood were no longer statistically significant at a 6-month follow-up. These results indicate that the majority of effects found immediately post-intervention were sustained in a later follow-up and demonstrates that, in some cases, impacts of the interventions were maintained for a period after the intervention ended. However, these results should be interpreted with caution as the length of follow-up time for the six studies varied from 4 weeks to 6 months and due to the small number of studies that included follow-up assessments the length of time decay for effects cannot be determined.

### Risk of bias assessment

Out of a total score of 5, the mean risk of bias score was 2.8 across the fourteen studies (see Supplementary Materials for full ratings). Of the fourteen studies, *n* = 1 study was rated as having low risk of bias (Sarkamo et al., 2013), *n* = 7 as moderate (Aleixo et al., 2022; Chen et al., 2018; Dawudi et al., 2023; Madsø et al., 2022; McDowell et al., 2023; Pongan et al., 2017; Tamplin et al., 2018), and *n* = 6 as high (Brotons & Marti, 2003; Camic et al., 2013; Holden et al., 2019; Jennings & Vance, 2002; Raglio et al., 2016; Satoh et al., 2015). One of the most common risks of bias was that the participants sampled were not deemed to be representative of the target population, largely due to low sample sizes of many of the studies. There was high inter-rater reliability with agreement of ratings reached on 87% of the appraisal items.

## Discussion

This review systematically synthesised existing evidence on the impacts of singing interventions in relation to quality of life, mood and agitation in community-dwelling people with dementia. Across 14 included studies, there was substantial variation in study and intervention design, with the majority of studies being quasi-experimental. The risk of bias across the studies was moderate to high indicating that the quality of the evidence was moderate to low. These characteristics are reflective of a field of research which needs more development, standardised methods and reporting. Nevertheless, the evidence can still be beneficial and inferences can be derived.

Across all of the included studies, despite some inconsistencies, evidence suggests that singing interventions led to a reduction in agitation levels and an improvement in mood in people with dementia. Evidence was found for this across both one-to-one and group singing interventions. This aligns with previous evidence that music interventions have a positive impact on mood (Lam et al., 2020) and levels of agitation (Kong et al., 2015). Evidence from this systematic review indicates that there is no robust evidence to suggest that singing interventions led to significant improvements in quality of life. This is consistent with some previous research that has also failed to find strong and robust evidence for impacts on quality of life following music interventions (Moreno-Morales et al., 2020; Sole et al., 2014). Nonetheless, this finding may be due to a lack of sensitivity in quality of life measures to detect subtle changes. This may also be due to a lack of studies which included a control group and general deterioration of dementia symptoms throughout the intervention may be masking improvements and effects of the singing intervention. For key developments in this area, future studies should strive to include a control group where possible which will allow for more robust examinations of singing interventions and allow intervention effects to be determined from general deterioration in dementia symptoms over time.

One-to-one and individualised singing interventions appeared to yield more consistent results than group singing interventions. Results indicated that one-to-one singing interventions may improve mood and depressive symptoms, and neuropsychiatric symptoms, with some studies indicating that these effects are sustained for a period of time after the intervention has ended (Holden et al., 2019). The tailored and individualised element that is incorporated into one-to-one singing interventions may be a key aspect of producing positive outcomes. Previous research comparing one-to-one and group music interventions found that participation levels were consistently higher in the one-to-one session compared to group sessions and it may be the case that this increased participation is a factor in producing greater impacts on mood and neuropsychiatric symptoms in people with dementia (Werner et al., 2017). Further, the majority of one-to-one singing interventions were delivered in the participant’s home as opposed to day centres, community centres and in some cases hospital settings for group interventions. This variation in setting may have impacted results and it could be that singing interventions delivered in familiar environments may produce more benefits than those delivered in unfamiliar settings.

The studies presented employed different intervention lengths ranging from 12 days to 18 months. Yet, from the results presented here, it cannot be determined what the optimal intervention length is for improvements in mood, quality of life or agitation. Due to the range of methods and outcome measures used across studies examining singing interventions, it is difficult to draw comparisons and discern essential parameters, structures and methods for best practice delivery of singing interventions likely to lead to consistent improvements in quality of life, mood and agitation. Despite a lack of conclusive evidence from this review, previous reviews that examined multiple types of music interventions in community-dwelling people with dementia (Hofbauer et al., 2022) found that longer interventions achieved fewer improvements than shorter interventions. It may be the case that longer-term singing interventions that become a more consistent and permanent aspect of peoples lives have a greater and longer-term impact compared to shorter interventions that have a defined end date when implemented. However, it should be noted that there were other key distinctions between short-term and long-term interventions with the majority of long-term interventions being delivered by caregivers rather than music therapists, researchers or clinicians and therefore it cannot be determined that the differences in results are solely due to intervention length (Hofbauer et al., 2022).

It is important to determine both the short-term and long-term impacts of singing interventions on agitation levels, mood and quality of life in people with dementia and particularly whether effects persist after the intervention has ended. Only a minority of studies included post-intervention follow-up assessments, with varying follow-up time periods (4 weeks to 6 months), however some findings indicated promising longer-term impacts on mood and neuropsychic symptoms post-intervention. Future research should include follow-up assessments after the end of the intervention to further determine the long-term effects of singing interventions and whether benefits persist. However, short-term improvements from attending singing interventions should not be overlooked. Short-term improvements in mood and agitation in people with dementia can be beneficial and can aid in improving carer relationships and interactions and ease care delivery to people with dementia. Improving carer interactions, particularly for people living within the community, could lead to positive benefits in the form of delaying the need for residential care and also positive improvements to the carer’s mental health and well-being.

### Limitations

There are some limitations of this systematic review, with one being moderate to high risk of bias ratings across the included studies. This reflects a field of research that needs further development with more consistent reporting and standardised methods. Additionally, only a minority of studies included follow-up assessments after the interventions ended and due to this, the long-term impacts of singing interventions cannot be fully determined in this review.

The majority of studies included in this systematic review did not include a control group in their study designs. As a result, it is difficult to discern intervention effects from general deterioration in dementia symptoms over time. For longer-term interventions, the need for a control group to be included in the study design is greater to be able to detect intervention effects from general deterioration of dementia symptoms likely to occur over time. However, the ethical and practical issues surrounding having a control group in such interventions should be acknowledged. By including a control group in these interventions’ researchers may be withholding or delaying a potentially beneficial intervention for people with dementia. These ethical and practical issues have likely contributed to the scarcity of control trials in the published research. Despite these limitations, this systematic review provides a useful indication of the effects of singing on community-dwelling people with dementia and highlights gaps in the literature in need of further development.

### Conclusions

Overall, this review provides evidence that community-based singing intervention may lead to a reduction in agitation and an improvement in mood and depressive symptoms. There was no strong evidence to suggest that singing interventions impacted the quality of life of people with dementia. This review highlights the potential of singing interventions as an effective psychosocial intervention for community-dwelling people with dementia, however, there is a lack of robust studies that have examined the impact of specifically singing interventions in community-dwelling people with dementia. This review highlights the need to determine the parameters required for singing interventions to have optimal benefits for people with dementia. Currently, it is unclear what works for singing interventions, in what situations and with whom. Future research is needed to delve into the exact parameters that are required to yield positive impacts on mood, quality of life and agitation in people with dementia. Future studies should focus on examining singing interventions with consistent outcome measures such as core outcome sets with the inclusion of a control group and follow-up assessments to determine longer-term post-intervention effects.

## Supplemental Material

Supplemental Material - The effects of singing interventions on quality of life, mood and levels of agitation in community-dwelling people living with dementia: A quantitative systematic review Supplemental Material for The effects of singing interventions on quality of life, mood and levels of agitation in community-dwelling people living with dementia: A quantitative systematic review by Megan Polden, Thomas Faulkner, Carol Holland, Kerry Hanna, Kym Ward, Faraz Ahmed, Heather Brown, Hazel Barrow, Jeanette Main, Stella Mann, Steve Pendrill, and Clarissa Giebel in Dementia

## Appendix 1

### Full search string

(((music* or Sing or sings OR singing or Singer* or song* or choir* or choral* or playlist* or listen* or concert*) and (dementia* or Alzheimer’s Disease* or Cognitive Impairment* or Mild Cognitive Impairment* or amnestic*) and (mood* or quality of life* depressive symptoms* or depression* or anxiety* or agitation*)))

### Abbreviations

DEMQOL: Dementia Quality of Life Scale
