# Supplementary material for: The effects of singing interventions on quality of life, mood and levels of agitation in community-dwelling people living with dementia: A quantitative systematic review
Source: Dementia (London). 2024 Aug 15;24(4):738–66. doi: 10.1177/14713012241273837 (PMC11997293; doi:10.1177/14713012241273837)
Supplement: Supplemental Material - The effects of singing interventions on quality of life, mood and levels of agitation in community-dwelling people living with dementia: A quantitative systematic review [file sj-pdf-1-dem-10.1177_14713012241273837.pdf]

Supplementary Materials: The effects of singing interventions on quality of life, mood and levels of agitation in community-dwelling people living with dementia: A quantitative systematic review

Table 1. Risk of bias scores and ratings using the Mixed Methods Appraisal Tool (Hong et al, 2018)

Author (date)	ROB score	Rating
Aleixo et al, (2022)	3	Moderate
Bronton, (2003)	2	High
Camic et al, (2013)	2	High
Chen et al, (2018)	4	Moderate
Dawudi et al, 2023	3	Moderate
Holden et al, (2019)	2	High
Jennings et al, (2002)	2	High
Madsø et al, (2022)	3	Moderate
McDowell et al, (2023)	3	Moderate
Pongan et al, (2017)	3	Moderate
Raglio et al, (2016)	2	High
Sarkamo et al, (2013)	5	Low
Tamplin et al, (2018)	3	Moderate
Satoh et al, (2015)	2	High

Note: ROB=Risk of bias. Risk of bias ratings out of a total score of 5
